# Style Transfer of Chinese Art Works Based on Dual Channel Deep Learning Model

**DOI:** 10.1155/2022/4376006

**Published:** 2022-09-20

**Authors:** Yan Tang

**Affiliations:** College of Art of Jiujiang University, Jiujiang, Jiangxi Province 332005, China

## Abstract

Aiming at the problems of style loss and lack of content in the style transfer of Chinese art works, this paper puts forward the style transfer technology of Chinese art works based on the dual channel deep learning model. On the basis of clarifying the technical principle of style transfer of art works, the image of art works is controlled and transformed based on the u-net network. The incomplete information in the restored image is filled, and the multiscale classification feature is used to calculate the color feature data items in the image. The sensitivity coefficient of color difference is calculated by using constraints, and the overlapping color discrimination and image segmentation of art images are realized. Poisson image editing is used to constrain the image spatial gradient to realize the style migration of art works. The experimental results show that this method can effectively avoid the problems of content error, distortion, and distortion in the process of art style migration, and has a better style migration effect.

## 1. Introduction

When appreciating the famous works of famous Chinese and foreign painters, we often hope to create a painting with a similar style. The emergence of image style transfer technology has helped people realize this wish [1]. People can convert any photo into any style of painting. In addition, in the era of the rise of a short video, various style filter effects are loved by people [2], and image style migration technology has been widely known by people. However, many image style migration methods can only target one style in a model, which is inefficient in an application.

Reference [3] proposes an example based image styling method based on the consistency of the target image during training. This method keeps the statistical data of neural response compatible with the data extracted from programmed sources and is widely used in video stylization, style conversion to panorama, face, and 3D models. Reference [4] mainly proposes improvements in the following two aspects: (1) adjust the structure of classical residual elements: convert the standard convolution into point convolution and depth convolution, and reduce the amount of computation while ensuring the convolution effect; (2) simplify the loss network: the fourth and fifth layers of the model are highly consistent in structure, and the effect of style restoration and content reconstruction of these two layers is basically the same, which ensures the effect of style restoration and content reconstruction while reducing the amount of parameters. Reference [5] proposed an unsupervised image style migration method based on an image mask. In the experiment, cyclegan architecture based on cyclic consistency is adopted, and a new generative model with a built-in image mask is designed by using the inception RESNET structure. Finally, the background of the image and the learned abstract features are automatically reorganized through unsupervised learning. Experiments show that the new method effectively separates and reorganizes the image background and abstract features, solves the problem of regional interference in the process of feature learning, and obtains considerable visual effect. Reference [6] in view of the difficulty of style extraction caused by the diversity of Mongolian clothing elements, large color differences, irregular patterns, and other characteristics, the method of combining K-means and a closed natural matting algorithm is used for image segmentation. The style and content of the image are extracted based on a neural network, and the resulting image is synthesized by image reconstruction technology to realize the style transfer of Mongolian and Chinese clothing image. Aiming at the problem of serious artifacts in the output image, an improved image style migration algorithm is adopted. Transform the input parameter of the image into a local constraint, which can suppress the distortion of the image. Aiming at the problem of spatial inconsistency in the style transfer of real photos, smooth it to ensure the consistency of spatial style after style processing. This method greatly speeds up the operation speed. Reference [7] proposed a periodic consistency antagonistic domain adaptive method with four input channels for vegetation region segmentation based on an index. This method preserves the specific ratio between near infrared and RGB bands and improves the segmentation network performance of the target domain. Reference [8] proposes a style library composed of multiple convolution filter banks, and each filter bank explicitly represents a style. In order to convert the image into a specific style, the corresponding filter bank is operated on the intermediate features generated by a single automatic encoder. This method can obtain the same results as the single parameter setting method.

Based on the application of image style transfer technology in interior decoration design based on the ecological environment in reference [9], this paper proposes the style transfer of Chinese art works based on the dual channel deep learning model. The art work style transfer technology proposed this time refers to converting the style of an art work into an image similar to the style of the art work through learning (using the dual channel deep learning model). This paper mainly puts forward improvements in the following two aspects: first, adjust the lighting of art works; Secondly, based on the dual channel convolution transformation and control of the u-net network, the incomplete information in the restored image is filled to ensure the effect of style restoration and content reconstruction of art works while reducing the amount of parameters.

## 2. Art Style Transfer Technology

Using different styles of art works to express the semantic content of images, so the method of image processing is the transfer of style of art works [10]. That is, on the premise of retaining the central content and structure of the original image, an ordinary image is displayed in another art work style. For example, there are two images, one is the style image and the other is the content image. The style transfer of art works is mainly divided into the following steps:

  Step 1: ensure that all the content and structure of the content image are preserved and then extract the main features of the style of the art work
  Step 2: use the extracted features to reconstruct the features of the original content image
  Step 3: perfectly combines the style of the style image and the content of the content image into a migration image, so as to obtain the output image

**Figure 1:**
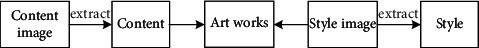
Flow chart of style transfer of art works.

The implementation process of the above process is shown in Figure 1.

### 2.1. Local Block Method of Double Threshold Image Based on Depth Convolution Neural Network

#### 2.1.1. Theoretical Analysis

Full convolutional networks (FCNs) is a network model based on deep learning, which is widely used in image segmentation. FCN has the following advantages:

1. The convolution network training is realized by replacing the full connection layer with the convolution layer.
2. In order to achieve pixel level segmentation, all pixel features in the image are predicted and classified. However, for the images with complex visual environment, the sampling on the FCN network structure still adopts the simplest deconvolution method, resulting in the inability to recognize the detailed features of the image, the final segmented image contour is blurred and the adhesion is serious.

Therefore, mask r-cnn is proposed as an instance segmentation method, and the region of interest (ROI) is taken as the network branch of the deep convolution neural network to realize the instance segmentation of the target image. In order to preserve the accuracy of the target spatial position coordinates, the mask r-cnn network replaces the roipool operation with the roialign operation. Roialign can correct the misplaced layers of spatial quantization feature extraction. The bilinear difference keeps the spatial position accuracy between the input network and the output network unchanged, corresponding to the coordinate value on the ROI bin. The dependence between the judgment class and the output mask is minimized, and the average binary cross entropy loss is used to predict the binary mask separately for each target. This process reduces the competitiveness between categories and improves the efficiency of image segmentation.

Based on the mask r-cnn network structure, the network depth and width are optimized and adjusted, and the migration learning is carried out on the given training parameters. Based on the segmented target image, the optimal network parameters and network model are obtained by calculating the segmentation accuracy between different layers and different convolution kernels.

#### 2.1.2. Local Blocking of Double Threshold Image

**Figure 2:**
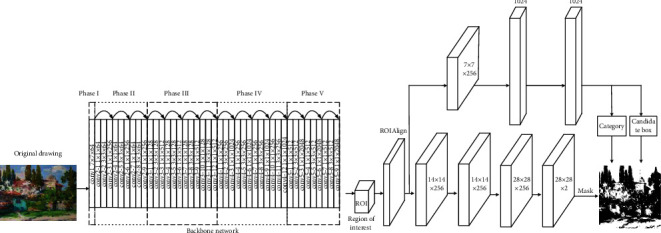
PigNet network structure.

Based on the above-given theoretical analysis, this paper determines the optimal network model as the Pignet network structure and makes two optimization improvements on the mask r-cnn network structure in terms of the number of convolution layers and categories:

1. For different target areas in the image, the fourth stage of mask r-cnn network changes from 69 convolution layers to 12 layers, which can reduce the feature loss on the one hand and the amount of convolution operation on the other hand.
2. The number of convolution layers in the last layer of the mask branch of mask r-cnn network is optimized and adjusted to Pignet and background. The specific structure is shown in Figure 2.

There are 44 convolution layers and five convolution layers, all of which adopt the Pignet structure. Each arc contains 3 convolution layers, 1 × 1 × 64 layers indicate that the convolution kernel is 1 × 1. Convolution layer with 64 channels. The residual learning structure reduces the number of parameters to a great extent, makes the calculation simpler and keeps the spatial position accuracy of the target unchanged. Through the arc part of the network diagram, the residual learning structure directly transmits the input information to the later layer, which also reduces some feature loss. The residual learning structure can also reduce the sliding step of each convolution layer from the original two pixels to one quarter, and the number of output channels increases continuously until 2048.

There are mainly two aspects of feature extraction in the Pignet backbone network structure: one is to analyze and process the feature map output by the network model conv4_12 convolution layer through the region proposal networks (RPN) [11] to extract the required feature information; on the other hand, it propagates forward to generate feature mapping. RPN can select the region of interest with the fastest speed.

The loss function *L* of the Pignet network is mainly composed of three parts: classification error *L*_cls_, detection error *L*_box_, and segmentation error *L*_mask_. The calculation formula is shown as follows:

(1) $$\begin{array}{c} L=L_{cls}+L_{box}+L_{mask}\text{.} \end{array}$$

Here, *L*_cls_ and *L*_box_ processes the full connection layer to predict the category of all regions of interest and the regression frame coordinate value of the target space. *L*_mask_ segment and mask the target image of each region of interest. Select the target image in which all regions of interest in the image are classified as pig, so that only the relative entropy error of pig needs to be considered when continuing to calculate the loss function generated by region segmentation. In order to avoid competition among classes, the background class is not considered when calculating the relative entropy error of pig class. *L*_box_ is mainly used to ensure that the position coordinates of the regression box of the target image do not deviate. *L*_mask_ is used to ensure the accuracy of the target image generation mask. Class branch predicts that the region of interest class is pig class, then *L*_mask_ only needs to predict pixels for pig class to ensure that the target image has clear contour and no adhesion, so as to ensure the accuracy of contour position coordinate information on different layer depths and realize accurate image segmentation. In this paper, the Pignet network model obtains two regions of interest from convolution calculation. *L*_box_ is used to predict the position coordinates of the target spatial regression frame. *L*_mask_ uses the combination of the average binary cross entropy loss function and sigmoid function to separately predict the position coordinates of the target spatial regression frame to form a binary mask. The segmented image is represented by two different color masks and placed in two different layer depths. Even if more images are segmented, the Pignet model will form a corresponding binary mask for each segmented target.

### 2.2. Extraction of Light Component in Non-significant Area

The multiscale Gaussian function can extract the illumination classification of different areas in the image by adjusting the adaptive parameters, so as to improve the quality of style migration of art works. The implementation process can be divided into the following steps:

  Step 1: image acquisition: obtain the image of the art work to be migrated
  Step 2: background removal: based on the HSI color space model, the *i*-component image is binarized according to the histogram selection threshold to form a binary mask image, and the two obtain the *i*-component image after removing the background through point multiplication
  Step 3: construct multi-scale Gaussian function filter
  Step 4: obtain the illumination component
  Step 5: perform homogenization correction on the surface brightness of the *i*-component image after removing the background

There are pixel structures with various structures in the art works. When extracting the illumination component in the nonsignificant area, the multiscale Gaussian function is used to set adaptive parameters in the image area to correct the empirical parameters existing in the art works. The correction process can be expressed as follows:

(2) $$\begin{array}{c} I\left(x,y\right)=I\frac{{\left(x,y\right)}^{\prime }}{\gamma \left(x,y\right)}\text{.} \end{array}$$

Here, *I*(*x*, *y*) represents the correction function formed, *γ*(*x*, *y*)′ represents the nonsignificant region extracted by bilateral filtering, and *γ*(*x*, *y*) represents the light source point function in art works. According to the image processing experience and the original illumination parameters in the nonsignificant area, the empirical value is selected. There are many illumination levels in the nonsignificant image area. In order to deal with the weakening effect of different light and dark areas on the nonsignificant area, the illumination points are randomly selected for adaptive adjustment. In order to eliminate the influence of image noise on image details, the image structure with a linear structure is selected to process the image after adaptive processing, and the structure parallel to the linear structure is selected to be processed as a corrosion reflection component to eliminate the noise in the image. The processing process is shown as follows:

(3) $$\begin{array}{c} R_{d}=\frac{R\;⊕\;H}{H}\text{.} \end{array}$$

Here, *R*_*d*_ represents the constructed denoising function, *R* represents the image expansion parameter, *H* represents the image structure parameter, and ⊕ represents the image panoramic processing. After eliminating the noise in the image, divide the front and back scenes in the image structure, take the back scene image as the extraction object of the light component in the nonsignificant area and extract the light component by using the adaptive gamma function [12, 13]. The extraction process is shown as follows:

(4) $$\begin{array}{c} a=-\frac{2R_{d}I\left(x,y\right)}{I_{\max}\left(x,y\right)}\text{.} \end{array}$$

Here, *a* represents the extracted parameters, *I*_max_(*x*, *y*) represents the maximum value of the image correction function, and the meaning of other parameters remains unchanged. In order to unify the dimension of the image illumination component, the above-extracted illumination component is linearly transformed by spatial image processing technology. The processing process is shown as follows:

(5) $$\begin{array}{c} g\left(x,y\right)=\frac{d-c}{b-a}\left[I\left(x,y\right)-a\right]\text{.} \end{array}$$

Here, *g*(*x*, *y*) represents the constructed linear function, *abc* and *d* represent the gray parameters of the illumination component, respectively, and the meaning of other parameters remain unchanged. After processing the illumination component in the image, the gray parameters in the image are divided according to the value size, and the gray level of small value is processed into a nonsignificant area [14]. For the image in this area, the image resolution is reconstructed by depth learning.

### 2.3. Poisson Image Editing Technology

The implementation process of Poisson image editing technology can be summarized as follows: assuming that the area occupied by the original image is Ω, according to the gradient of the original image and the boundary structure of the embedding position of the target image, the image pixel value of the transition region is constructed and seamlessly fused to the right image. The image value of the right fusion part is unknown, and the energy function needs to be solved. That is, for an image, the macro significance is reflected in the texture features, so to make the two images integrate properly and seamlessly, it is necessary to make the texture of the fused part of the two images consistent. The texture is reflected in the gradient, so the gradient of the fused region should be consistent with that of the original image. The consistency of fused textures means that the variation difference between the inner and outer regions of the original image reaches a minimum; that is, the energy function takes a minimum.

**Figure 3:**
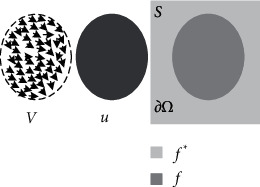
Schematic diagram of image pixel construction in fusion area.

The image used for the style transfer of art works is generally a fuzzy image. Logically, a fuzzy image is an image in which the contour of the object is not obvious and the gray change of the contour edge is not enough, resulting in a weak sense of hierarchy. In order to generate a clear image, we need to calculate the change rate of the image gray level and then obtain a clearer style transfer image. In the style transfer of art works, we should also pay special attention to not making the whole decoration look ambiguous. We should find the “change rate of image gray level” and then successfully fuse the source image with the target image. The image pixel construction diagram of the fusion area is shown in Figure 3.

In Figure 3, *V* represents the gradient field of the source image, *u* represents the source image of the art work, *∂*Ω represents the boundary of the image, *f*^*∗*^ represents the result of the combination of images other than Ω, Ω represents the area covered by the image, and *f* represents the image within Ω.

## 3. Realize the Style Transfer of Art Works

**Figure 4:**
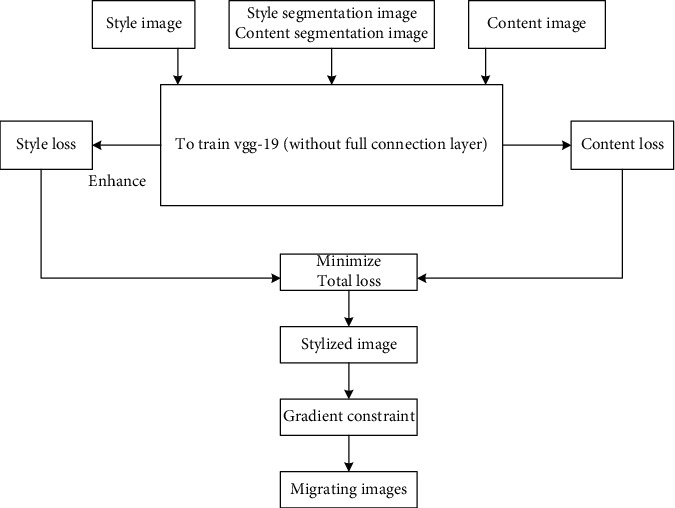
Style transfer process of art works.

Due to the authenticity and complexity of the content in the style transfer process of art works, it is necessary to keep the consistency of the content image and the style as much as possible in the transfer process, so that there will be no error or distortion in the style transfer image content of art works [15, 16], which makes the effect after the transfer real and effective. The implementation process of style migration is shown in Figure 4.

According to the migration process shown in Figure 4, the migration process is analyzed in detail.

### 3.1. Image Segmentation of Art Works

The image segmentation technology of art works mainly through point by point convolution and pool layered recognition. After a series of processing of the image of art works, the final output feature vector of the image is clear and reliable, which is helpful to accurately distinguish the image category. Although the loss of pixels limits the pixel level classification, it does not affect the two-dimensional spatial information of the image. At this time, the full connection layer can be converted into a convolution layer, the original size of the image can be restored through deconvolution operation, and then each pixel can be classified one by one to realize the effective segmentation of the image of art works. This method is not limited by the size of the input image, reduces the repeated storage and convolution calculation of pixel blocks to a certain extent and improves the extraction efficiency of image features. In terms of data sets of different sizes, the image segmentation based on the u-net network [17, 18] has a good segmentation application effect.

The specific features of the u-net network include the following:

1. The image full connection layer can be transformed into a convolution layer in real time. The number of image convolution results output by the last convolution layer is equal to the number of image classification. For the image of art works, the parameter value of each coordinate point represents the probability corresponding to the pixel point and the relevant category, and the category to which the image feature belongs is the largest corresponding probability value.
2. When the input data in the image of art works passes through the pool layer, the output image features will be reduced to a certain extent. The size of the feature image obtained by the last down sampling will be reduced to 1/16 of the original image. On this basis, the restored image can be obtained by adding the deconvolution layer, recovering with the upper sampling, and fusing with the image after the change of the expansion path, and the size is the same as the original image.
3. The jump structure is added to the u-net network to extract the detailed features in the image of art works. At the same time, the semantic features are extracted through the network. After splicing and fusing the multiscale features, the image with complete and rich information is obtained.

Therefore, the u-net network is used for image segmentation of art works. The image of art works will produce color overlap in some special states. At this time, if only the color features in the image are used to judge the image information, it is easy to produce inaccurate boundary segmentation. Therefore, the data items in the image are calculated by using the multiscale classification features [19, 20].

(6) $$\begin{array}{c} D\left(a_{u},\theta \right)=-1n\left(\gamma p_{c}\left(v_{u}^{\prime }\left|a_{u}\right.\right)+\left(1-\gamma \right)p_{s}\left(v_{u}^{\prime }\right)\right)\text{.} \end{array}$$

Here, *Vu*′ represents the *u*-th color vertex in the adjacent pixel area in the target image, *a*_*u*_ ∈ {0,1} represents the correlation label between the foreground and the background in the image of art works, *p*_*c*_(*v*_*u*_′|*a*_*u*_) represents the constraint term of the color in the image background, *p*_*s*_(*v*_*u*_′) represents the constraint term of the multi-scale classification feature on the image color, and *γ* represents the adjustment coefficient of the constraint term.

When the colors in the images of art works overlap, the multiscale classification feature plays a decisive role in the classification of graphics. Therefore, generally, the value of the adjustment coefficient *γ* will be relatively small. The specific calculation process is shown as follows:

(7) $$\begin{array}{c} \gamma =\left|P_{c}\left(v_{u}^{\prime }\left|1\right.\right)-P_{c}\left(v_{u}^{\prime }\left|0\right.\right)\left|{}^{\beta }\left(\beta \in \left[0,1\right]\right)\right.\right.\text{.} \end{array}$$

Here, *β* is the sensitivity coefficient to adjust the main color difference of the image, and the general value is set at 0.5. By calculating the shortest Euclidean distance between the vertex *Vu*′ and the foreground seed and background seed in the image [21, 22], the degree of overlap of the main color distribution in the image can be obtained. The calculation method of Euclidean distance is shown in formulas (8) and (9):

(8) $$\begin{array}{c} d_{u}^{F}=\min\left\|I_{u}^{\prime }-\theta_{F}\right\|\text{,} \end{array}$$

(9) $$\begin{array}{c} d_{u}^{B}=\min\left\|I_{u}^{\prime }-\theta_{B}\right\|\text{.} \end{array}$$

In formulas (8) and (9), *θ*_*F*_ and *θ*_*B*_ represent the color values of foreground seed and background seed, respectively, and *Iu*′ represents the vertex color value. After obtaining the above data, we can further obtain the color constraint value of the image of art works, as shown in the following formula:

(10) $$\begin{array}{c} P_{c}\left(v_{u}^{\prime }\left|a_{u}\right.\right)=\left\{\begin{array}{c} \frac{d_{u}^{B}}{d_{u}^{F}+d_{u}^{B}},ifa_{u}=1,andd_{u}^{F}+d_{u}^{B}\neq 0\text{,}\\ \frac{d_{u}^{B}}{d_{u}^{F}+d_{u}^{B}},ifa_{u}=0,andd_{u}^{F}+d_{u}^{B}\neq 0\text{,}\\ 0.5,ifd_{u}^{F}+d_{u}^{B}=0\text{.} \end{array}\right. \end{array}$$

The multiscale typing feature constraint is mainly determined by the eigenvalue *S*_*MP*_. By adjusting the level variable eigenvalues in the foreground and background, the corresponding multiscale typing features are inversely increased. In this process, the adjustment is completed by adjusting the variable factor and the feature mean value is obtained. At this time, the foreground multi-scale typing eigenvalues, as shown in the following formula:

(11) $$\begin{array}{c} {S^{\prime }}_{Fu}=\frac{P_{c}\left(v_{u}^{\prime }\left|a_{u}\right.\right)\times S_{MF}^{2}}{m}\text{.} \end{array}$$

Here, *m* represents the mean value of typing characteristics. The calculated characteristic values of background multi-scale classification, as shown in the following formula:

(12) $$\begin{array}{c} {S^{\prime }}_{Bu}=\frac{{\left(1-S_{Fu}^{\prime }\right)}^{2}}{1-m}\text{.} \end{array}$$

The calculation formula of typing characteristic mean *m*, as shown in the following formula:

(13) $$\begin{array}{c} m=\frac{1}{n}\overset{n}{\underset{u=1}{\sum }}{S^{\prime }}_{Bu}\text{.} \end{array}$$

The constraint process of multi-scale segmentation between image foreground and background, as shown in the following formula:

(14) $$\begin{array}{c} \left\{\begin{array}{c} p_{s}\left(v_{u}^{\prime }\in F\right)=\frac{{S^{\prime }}_{Fu}}{{S^{\prime }}_{Fu}+{S^{\prime }}_{Bu}}\\ p_{s}\left(v_{u}^{\prime }\in B\right)=\frac{{S^{\prime }}_{Bu}}{{S^{\prime }}_{Fu}+{S^{\prime }}_{Bu}} \end{array}\right.\text{.} \end{array}$$

In order to effectively improve the segmentation accuracy of the image of art works, the image area with a relatively high classification eigenvalue of the area with a relatively small area shall be subject to unified noise reduction. If *p*_*s*_(*v*_*u*_′ ∈ *F*)=0.5 and *A*_*u*_ < *A*_avg_ conditions are met, then *p*_*s*_(*v*_*u*_′ ∈ *F*)=0.5 and *A*_avg_ represent the average area of the image. So far, the image segmentation of art works is completed.

### 3.2. Content Loss

For the image of art works, the style image is defined as *s*, the content image as C and the white noise image [23, 24], and it is input into the vgg-19 network to extract the style of the interior decoration art image through the low-level response, extract the content of the style migration image of art works through the high-level response and use the random white noise image as the initial input. In this way, we can make up for the deficiency of content feature map and white noise feature map, get many feature maps under the action of the convolution layer, and convert conv3_ 2, conv4_ The second layer is described as the content image of the style transfer of art works. An image is generated in the style transfer of art works, which is similar to the content image C in content design. The average loss function is introduced into the calculation of content loss, and the calculation formula, as shown in the following formula:

(15) $$\begin{array}{c} L_{coabut}\left(c,g,l\right)=\frac{1}{2}\sum {\left\|K_{l}\left(g\right)-K_{l}\left(c\right)\right\|}^{2}\text{.} \end{array}$$

Here, *l* represents the convolution layer and *K*_*l*_ represents the feature matrix of image *g* in the style transfer of art works.

Using the theory of error direction propagation, the gradient value of the generated image *g* in the style migration of art works is calculated and updated to the input image, so that the initial random image changes until the same response as the content image *C* appears in the style migration network of art works.

### 3.3. Enhanced Style Loss

Image style is expressed by the correlation between features. Art work style is texture information, which refers to calculating the relationship between features through Gram matrix, so as to capture the texture information of art work style migration image, and through conv2_1, conv1_1, conv4_1, conv3_1 conv5_1 as the style of the image, the gradient descent mode of the white noise image [25, 26] is used to match the image similar to the style of the art work. In addition, mark the segmented image in another channel and take it as the input mode to form the style loss for each semantic category. Use the segmented channel to enhance the algorithm of the convolutional neural network and form the style loss between the output image *g* and the style images through the calculation of function:

(16) $$\begin{array}{c} L_{style}\left(s,g,l,c\right)={\overset{c}{\underset{c=1}{\sum }}\frac{1}{2}\sum \left\|G_{c,l}\left(g\right)-G_{c,l}\left(s\right)\right\|}^{2}\text{.} \end{array}$$

Here, *G*_*c*,*l*_ represents the operation mode of Gram matrix, that is, the inner product between the style transfer graphs of art works and *c* represents the number of semantic segmentation mask categories in the style transfer of art works.

The loss function of art style migration image, as shown in the following formula:

(17) $$\begin{array}{c} L_{total}=\alpha L_{content}+\beta L_{style}\text{.} \end{array}$$

After the minimization iteration of the loss function, the stylized image of art style transfer is obtained.

### 3.4. Poisson Image Editing Constraint Image Spatial Gradient

In order to generate a clear style transfer effect in the style transfer of art works, take the style transfer image of art works after style processing as the input, then the gradient field of the content image can be expressed, as shown in the following formula:

(18) $$\begin{array}{c} g\left(x,y\right)=\nabla c\left(x,y\right)\text{.} \end{array}$$

While constraining the image spatial gradient, it also needs to meet the following requirements, as shown in the following formula:

(19) $$\begin{array}{c} L^{*}=\min\int \int_{\Omega }{\left\|\nabla F-g\right\|}^{2}+{\left(F-C_{s}\right)}^{2}\text{.} \end{array}$$

Based on the objective function of spatial gradient constraint, the Poisson equation is established, as shown in the following formula:

(20) $$\begin{array}{c} F\left(1-\lambda \nabla^{2}\right)=C_{s}-\lambda \nabla g\text{.} \end{array}$$

Here, *λ* represents the relative weight between the control content image and the style image.

To sum up, the style transfer steps of art work style transfer image are summarized as follows:

  Step 1: input the art style migration style image, art style migration content image, and their segmented images into the trained vgg-19 network, initialize the white noise map of image pixels, and input them into the vgg-19 network together.
  Step 2: conv3 in vgg-19 network_ Layer 2 and conv4-2, extract the content feature matrix of the art work style migration content image and calculate the content loss value between the image pixel white noise image and the content image.
  Step 3: conv1 in vgg-19 network_ 1st floor, conv2-1 floor, conv3_ Layer 1, conv4-1, and conv5-1, extract the style feature matrix of the style transfer image of the art work, take the color marked segmented image as another channel of the style transfer of the art work, connect all the segmented channels, and calculate the enhanced style loss value of the image pixel white noise image and the style image.
  Step 4: calculate the total loss function in terms of content loss and style loss of the style migration image of art works used for training.
  Step 5: by training the style transfer image of art works, the white noise gradient of image pixels can be reduced, so as to minimize the total loss function. After several iterative calculations, the total loss function is adjusted to obtain the stylized image of interior decoration art; Poisson image editing technology is used to constrain the gradient of stylized images, and the migration effect picture of image content with both art work style migration style and art work style migration content is obtained.

According to the above process, complete the style transfer of art works.

## 4. Experimental Analysis

### 4.1. Experimental Data Set

In order to verify the practicability of the proposed style transfer method, 2200 art works of different styles are randomly selected from 15 groups of open art image data sets as pretraining data sets. And, input it into vgg-19 network model for pretraining to obtain model parameters. After setting the name of the image in 15 groups of open image data sets of art works, sort out the parameters of art works, as shown in Table 1.

Using the data set of art works shown in Table 1, applying the parameters in the above table, the median difference method is used to calculate the regional gradient in the image. The numerical, as shown in the following formula:

(21) $$\begin{array}{c} \left\{\begin{array}{c} G_{x}\left(i,j\right)=\frac{\left[V\left(i+1,j\right)-V\left(i+1,j\right)\right]}{2}\\ G_{y}\left(i,j\right)=\frac{\left[V\left(i+1,j\right)-V\left(i+1,j\right)\right]}{2}\\ gr\left(V\left(i,j\right)\right)=\sqrt{G_{x}{\left(i,j\right)}^{2}} \end{array}\right.\text{.} \end{array}$$

Here, *V*(*i*, *j*) represents the pixel function of the art work, *gr*(*V*(*i*, *j*)) represents the amplitude value of the art work at the position of pixel (*i*, *j*), *G*_*x*_(*i*, *j*) represents the horizontal gradient value of the image, and *xmeG*_*y*_(*i*, *j*) represents the vertical gradient of the image. After the enhanced image is processed into regional gradient values, the gradient parameters in the data set are sorted out as the processing object, and the application performance of the proposed method is tested with reference [5] unsupervised image style migration technology based on image mask and reference [6] image style migration technology based on semantic segmentation.

### 4.2. Results and Analysis

**Figure 5:**
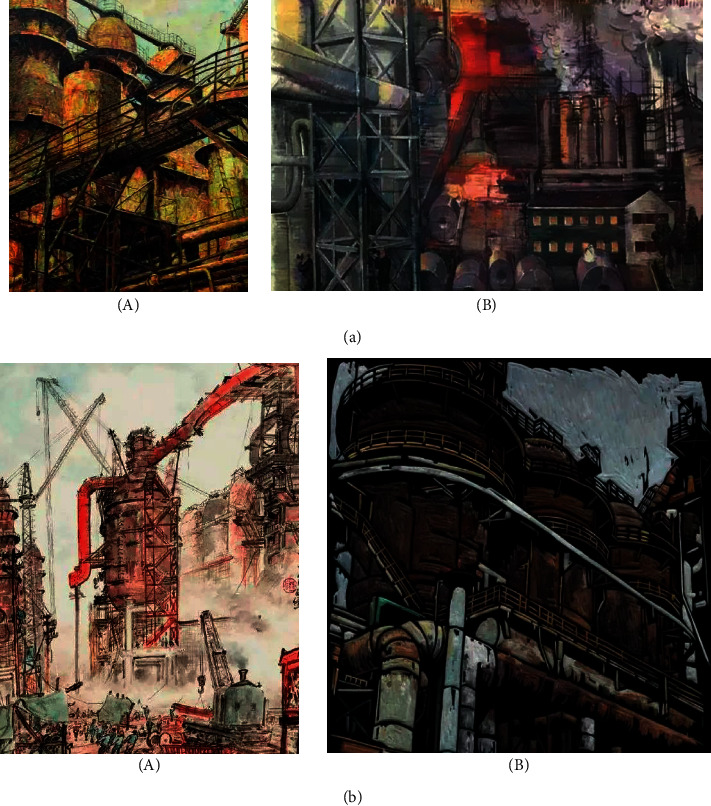
Style transfer of art works style transfer effect of art works (a) Premigration image (b) Post migration image.

In order to test the robustness of the proposed art work style migration technology, two art work scenes with different scenes are selected, and each scene selects different style types for migration and conversion. The results are shown in Figure 5. (from the Internet).

It can be seen from the migration results in Figure 5 that the content image corresponding to scene (a) belongs to the style of “lining people with objects,” and the corresponding style image belongs to the realistic style. A good migration effect can be generated between the content image and the style image; scene (b) is to test the style conversion effect of style migration images of art works with the same style and different contents. The corresponding style images and content images belong to “lining people with objects,” but they can still produce a good migration effect.

Based on the above-given experimental preparation, the distortion parameters after image migration are defined by using the parameters obtained by the gradient processing; the numerical relationship is shown as follows:

(22) $$\begin{array}{c} D\left(v_{1},v_{2}\right)=\sqrt{{\left(v_{1},v_{2}\right)}^{T}\left(\frac{\sum_{1}+\sum_{2}}{2}\right)}\text{.} \end{array}$$

**Figure 6:**
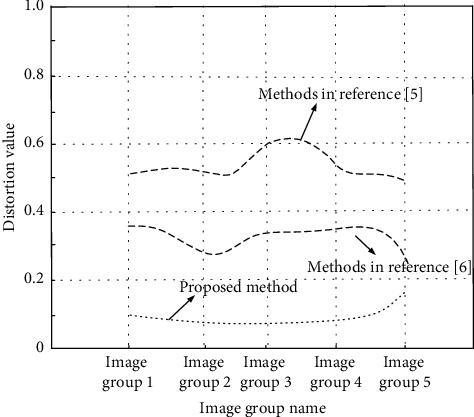
Distortion parameters generated by three methods.

Here, *v*_1_ and *v*_2_, respectively, represent the distortion vector of the image, Σ_1_ and Σ_2_, respectively, represent the covariance value of the image, *T* represents the multivariate parameter in the image, and *D*(*v*_1_, *v*_2_) represents the image distortion parameter. Corresponding to the numerical relationship constructed above, every three sets of art works data sets are processed into an independent variable. Finally, the image distortion parameter results after migration by the three methods are shown in Figure 6.

It can be seen from the experimental results shown in Figure 6 that 15 groups of open source images are integrated into 5 groups of image processing groups. Corresponding to the distortion parameter value relationship constructed above, the greater the calculated distortion value is defined, it means that the migrated image of this style migration technology has greater distortion. According to the values in Figure 6, the average distortion parameter obtained by the method of reference [5] is about 0.55, and the migrated image has great distortion. The average distortion parameter obtained by the method in reference [6] is 0.3, and the image migrated by this style migration technology produces less distortion. The average distortion parameter obtained by the proposed method is 0.15. Compared with the two comparison methods, the distortion parameter formed by the proposed style migration technology is the smallest, and the distortion generated by the actual migrated image does not affect the next image processing work.

In the above-given experimental environment, three methods are called to segment the nonsignificant region, and multiple overlapping measures are defined in the three methods. In the actual segmentation, when there is no overlap in the overlapping measures, it indicates that the image style migration technology can accurately segment the nonsignificant region, and the overlap degree of the three methods is defined. The numerical relationship is shown as follows:

(23) $$\begin{array}{c} I=\frac{A\times V\left(i,j\right)}{B}\text{.} \end{array}$$

**Figure 7:**
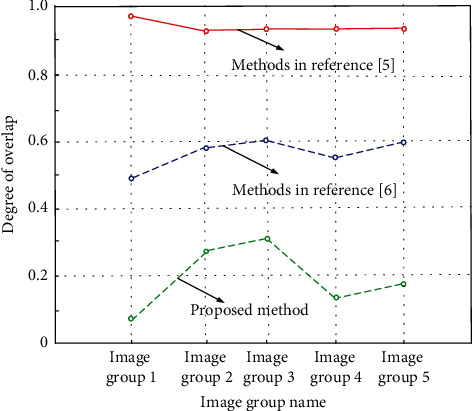
Overlap values generated by the three methods.

Here, *I* represents the calculated overlap, *A* represents the significant area segmented by image style transfer technology, and *B* represents the nonsignificant area segmented by style transfer technology. Under the control of the above numerical relationship, sort out the art works data set prepared for the experiment, and sort out the overlap results generated by the three methods, as shown in Figure 7.

After the images participating in the experiment are divided into five groups of image groups, the average value of overlap in the image group is calculated according to the above-constructed overlap value relationship. It is defined that the closer the overlap value is to 1, it means that the significant area and nonsignificant area segmented by this style of migration technology overlap. According to the numerical relationship shown in Figure 7, the average overlap value obtained by the method of reference [6] is 0.9, There is a lot of overlap between the nonsignificant region and the significant region segmented by this style transfer technology, which has a great impact on enhancing the nonsignificant region. The average overlap obtained by the method of reference [5] is 0.5. The nonsignificant region segmented by this style of migration technology overlaps with the significant region, which has little impact on the process of image migration. The average overlap obtained by the proposed method is 0.2. Compared with the two comparison methods, the overlap between the nonsignificant region and the significant region actually segmented by the proposed method is small, which has little impact on the effect after migration.

**Figure 8:**
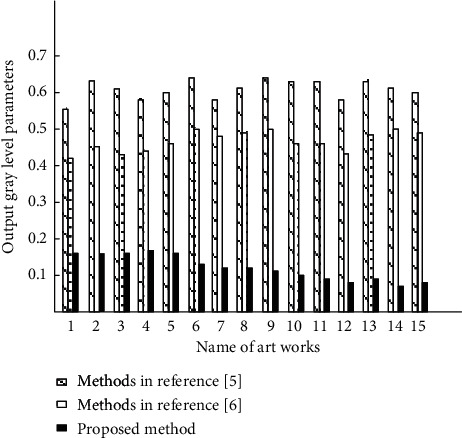
Gray level parameters output by three methods.

Keeping the above-given experimental environment unchanged, the brightness of the art works processed by the three methods is stretched and processed into a light compensation process. After the stretching process of the same process, the gray level output by the three methods corresponds to the gray level output. The output gray level results are shown in Figure 8.

After the same illumination treatment, the art works prepared for the experiment are marked in order by comparing the gray level parameters of the output of the actual stretching enhanced image. It is defined that the larger the gray level parameter of the image output after migration, it indicates the loss of details of the image area after illumination compensation. According to the gray level parameter values sorted and calculated in Figure 8, when referring to the art works after migration according to the method of reference [6], the actual output gray level parameter is between 0.5 and 0.7, the actual output gray level parameter is the largest, and the nonsignificant area after migration loses the most details. The actual output gray level parameters of reference [5] method are between 0.4 and 0.5, the actual output gray level parameters are large, and the nonsignificant areas after migration lose more details. The actual output gray level parameters of the proposed method are between 0 and 0.2. Compared with the two comparison methods, the output gray level parameters are the smallest, the details of the nonsignificant area of the migrated image are the least, and the quality of the migrated image is the best.

In conclusion, the experimental results prove the effectiveness of the proposed method. In the above tests, the average distortion parameter obtained by the proposed method is 0.15, the average overlap is 0.2, and the actual output gray level parameter is between 0 and 0.2. The test results of the three test indicators are low, which shows that the proposed art work style migration technology has very strong robustness and can achieve art work style migration. And this method can not only achieve a better migration effect but also will not make the style migration image of art works distorted.

## 5. Conclusion

Art works are an important tool of expression and communication in daily life and social life. However, with the changes of the times and the evolution of culture, the functions of the style of art works in reflecting cultural heritage, emotional appeal, visual beauty, as well as brand image, product information, industry characteristics, etc., have attracted more and more attention. Especially in recent years, with the vigorous development of digital carriers, digital design of art works has gradually become a daily demand of today's society and life. Therefore, aiming at the problems existing in the style image of art work style transfer, this paper proposes a style transfer technology of art work based on dual channel deep learning. Using the principle of art style migration technology and Poisson image editing technology, the style migration of art works is realized, so as to obtain a migrated image that can meet the modern style design. The experimental results show that the proposed method has very strong robustness and can realize the style migration of art works. It can not only achieve a better migration effect but also will not make the style migration image of art works distorted.

## Figures and Tables

**Table 1:** Parameters of art works data set prepared.

Artwork dataset name	Positive sample (%)	Size	Resolution (dpi)
Labeled faces in the wild	48273 (7.64)	640 × 480	100 million
MNIST-06	39390 (39.8)	1280 × 640	20 million
CIFAR-02	36640 (8.21)	640 × 480	100 million
AI-challneger-04	63605 (40.2)	1280 × 640	20 million
Pascal VOC-07	27001 (47.21)	640 × 480	100 million
COCO common objects dataset	75220 (4.1)	1280 × 640	20 million
CityScapes	51220 (46.9)	640 × 480	100 million
Lego bricks	19090 (38.67)	640 × 480	100 million
Visual genome	75904 (17.62)	640 × 480	100 million
VisualQA	72490 (25.05)	1280 × 640	20 million
MNIST-08	36257 (47.14)	640 × 480	100 million
KITTI-05	28284 (30.13)	1280 × 640	20 million
ApolloScape-02	33298 (18.37)	1280 × 640	20 million
TUM-08	68273 (1.9)	640 × 480	100 million

## Data Availability

The raw data supporting the conclusions of this article will be made available by the author, without undue reservation.
